# Hearing Impairment in Kazakhstan, 2020–2025: Dispensary Registration, Disability Certification, and Cochlear Implantation Response, with Reference to Global Burden of Disease 2021 Modelled Estimates

**DOI:** 10.3390/audiolres16040104

**Published:** 2026-07-24

**Authors:** Zhadra Bukenova, Galiya Orazova, Aigerim Baimagambetova, Zhadyra Karashutova, Aisulu Talgatova, Zulfiya Zholdybayeva, Sauran Yerdessov, Gulnara Kulkayeva

**Affiliations:** 1 Department of Epidemiology and Biostatistics, Astana Medical University, Astana 010000, Kazakhstan; zhadrabukenova777@gmail.com; 2 School of Public Health and Management, Astana Medical University, Astana 010000, Kazakhstan; 3 National Scientific Center for Health Development Named After Salidat Kairbekova, Astana 010000, Kazakhstan; 4 Department of ENT Diseases, Astana Medical University, Astana 010000, Kazakhstan; 5 Department of Medicine, Nazarbayev University School of Medicine, Astana 010000, Kazakhstan

**Keywords:** hearing impairment, dispensary registration, healthcare utilisation, disability, cochlear implantation, Kazakhstan, Central Asia, cascade of care, Global Burden of Disease

## Abstract

**Background:** Hearing health in Kazakhstan is documented through parallel administrative systems whose indicators are often conflated. We examined three administrative layers of hearing health during 2020–2025: dispensary registration of ear and hearing-loss diagnoses, disability certification as a severe administrative endpoint, and cochlear implantation (CI) as the specialised treatment response, with reference to Global Burden of Disease (GBD) 2021 estimates. **Methods:** We conducted a national ecological, region-year, multi-source administrative registry study across all 20 regions of Kazakhstan from 2020 to 2025. The dataset included 114 region-year observations and integrated Dispensary Report Form No. 12, the State Register of Persons with Disabilities, and the Electronic Register of Inpatient Patients. Dispensary indicators were interpreted as registered healthcare-utilisation measures, not population prevalence or incidence. Trends were assessed using Mann–Kendall tests with Benjamini–Hochberg false-discovery-rate correction. Regional patterns were examined using Spearman correlation, inequality metrics, and exploratory clustering. **Results:** Among adults, registered H60–H95 annual morbidity increased from 231,209 to 278,897 cases (CAGR +3.8%; FDR *p* = 0.016), and first-time registrations rose from 126,513 to 167,674 (CAGR +5.8%; FDR *p* = 0.016). Active dispensary observation declined modestly from 21,840 to 19,881. H65–H66 chronic otitis first-time registration remained stable, while active observation declined sharply. Paediatric H90–H91 registration was stable. In 2025, 31,560 persons were registered with hearing impairment-related disability, including 26,522 adults. CI procedures increased from 285 to 523. Adult CI activity was intermittent between 2012 and 2018 (peak 140 in 2013), ceased in 2019–2020, and was re-established from 2021 (14 → 129 procedures in 2025), reaching 24.7% of all CI. Most procedures were concentrated in Almaty and Astana. Compared with the GBD-2021 estimate of 17,212 per 100,000, administrative indicators captured much narrower service-utilisation and severe-certification layers. **Conclusions:** Kazakhstan’s administrative data describe distinct hearing-health layers rather than competing prevalence estimates. Adult screening, regional audiology capacity, post-CI rehabilitation, and longitudinal outcome registries are priority areas.

## 1. Introduction

Hearing loss ranks among the most prevalent sensory impairments worldwide and is the third leading cause of years lived with disability (YLDs) [1]. The Global Burden of Disease (GBD) 2021 study estimates that approximately 1.5 billion people live with hearing loss at the audiometric threshold of ≥20 dB HL in the better-hearing ear, of whom about 430 million meet the World Health Organisation (WHO) definition of disabling hearing loss (>35 dB HL) [1,2]. The economic burden of unaddressed hearing loss is substantial globally, with WHO estimating annual costs of approximately US$980 billion [3]. The WHO has identified hearing care as a global public health priority [4]. Untreated adult hearing loss is associated with cognitive decline and incident dementia [5,6], depression [7], hospitalisation [8], and increased mortality. Cochlear implantation (CI) provides effective rehabilitation for severe-to-profound bilateral sensorineural hearing loss in both children and adults [9,10], but global access remains highly uneven [11].

In Kazakhstan, an upper-middle-income country of approximately 20 million people in Central Asia, several administrative systems document hearing health in parallel: dispensary morbidity reporting via Statistical Form No. 12, the State Register of Persons with Disabilities, and the Electronic Register of Inpatient Patients for high-cost interventions including CI. Each of these systems answers a different question about the population. Dispensary reporting captures who arrives at the formal healthcare system with an ear or hearing diagnosis. Disability certification captures who is recognised by the medico-social expert evaluation (MSEK) framework as having a functional limitation severe enough to warrant social support. The CI register captures who receives high-technology rehabilitation. None of these is a measure of true population prevalence in the sense used by the GBD study, which models the audiometric burden in the general population [1].

Conflating these layers—for example, treating dispensary observation counts as prevalence, or treating disability certification rates as a measure of how much hearing loss exists in the population—can produce misleading estimates of need. We therefore report each layer under its own definition: dispensary indicators as healthcare-utilisation measures, disability registration as a certified severe-impairment indicator, and cochlear implantation as a treatment-response indicator. The GBD 2021 modelled estimate is used as an external reference frame rather than as a directly competing prevalence measure. Importantly, the disability registry and the dispensary system are parallel administrative tracks rather than strict subsets of one another: a person can be certified as disabled without being under active dispensary observation, and a person under dispensary care may never meet disability-certification thresholds.

Kazakhstan-specific GBD 2021 analysis estimates an age-standardised prevalence rate of hearing loss at 17,212 per 100,000 population, with a YLD rate of 512 per 100,000 [12]. Cross-sectional audiometric screening in Astana detected hearing loss in 20% of adults, with 71.9% in the mild category (20–34 dB HL) [12]. A recent regulatory development—Order No. 92 of the Minister of Health (12 September 2025)—for the first time extends state-funded hearing aid provision to non-disabled adults with hearing loss [13], creating a regulatory pathway for population-level intervention beyond the disability framework. Despite these foundational data, no comprehensive multi-source national analysis of administrative hearing-health registration patterns has been published in the international peer-reviewed literature.

This study therefore aims to describe the Kazakhstani hearing-health administrative landscape for 2020–2025 by sequentially examining each administrative layer: (i) dispensary registration of ear and hearing diagnoses, with explicit disambiguation of registered annual morbidity, first-time-in-life registration, and active dispensary observation, separately for the broad H60–H95 category, the H65–H66 chronic otitis category, and the H90–H91 specific hearing-loss category; (ii) disability certification as the severe administrative endpoint; (iii) cochlear implantation as the specialised service response, distinguishing adult and paediatric uptake and quantifying geographic access; and (iv) a final section that places these administrative layers in relation to the GBD-2021 modelled audiometric estimate as an external reference frame.

## 2. Materials and Methods

### 2.1. Study Design and Setting

We conducted a national ecological, region-year, multi-source administrative registry study covering all 20 administrative regions of the Republic of Kazakhstan, including 17 oblasts and three cities of republican significance: Astana, Almaty, and Shymkent, over six calendar years from 2020 to 2025. Following the 2022 administrative reform, three new oblasts (Abai, Zhetysu, and Ulytau) were established from East Kazakhstan, Almaty region, and Karaganda. Pre-2022 population denominators were harmonised to the administrative structure used in the source datasets, and sensitivity analyses excluding reform-affected regions were performed. The final analytical dataset comprised 114 region-year observations × 111 variables—six fewer than the theoretical maximum of 120 (20 × 6) because the three new oblasts did not exist in 2020 or 2021. Reporting follows the STROBE [14] and RECORD [15] guidance.

### 2.2. Data Sources

Three national administrative sources were merged into a single master analytical dataset.

(1) **Dispensary Report Form No. 12**: annual regional aggregate reports on disease registration, disaggregated by age group (children 0–14 years, adolescents 15–17 years, adults ≥ 18 years) and sex, for ICD-10 codes H60–H95 (diseases of the ear and mastoid process—broad category), H65–H66 (otitis media—chronic suppurative and non-suppurative), and H90–H91 (conductive, sensorineural, mixed, and unspecified hearing loss). H90–H91 is reported separately only for children in the dispensary form; for adults, hearing-loss-specific cases are aggregated within H60–H95.
(2) **State Register of Persons with Disabilities:** annual cross-sectional counts of persons holding active disability status as of 1 January each year, stratified by region, age group (adults ≥ 18/children 0–17), sex, and place of residence. Disability classifications included the broader H60–H95 category, the specific ‘hearing impairment defect’ category, and the combined ‘hearing impairment with deaf-mutism’ category. Disability status is granted by the medico-social expert evaluation (MSEK) commission. For adults, certification typically requires fourth-degree (grade IV) bilateral hearing loss not amenable to hearing aid correction, or complete deafness; for children, grade III bilateral hearing loss is sufficient.
(3) **Electronic Register of Inpatient Patients**: records of cochlear implantation procedures by performing facility, region, year, and recipient age group (adult ≥ 18/child < 18). The age-stratified records—newly available in this analysis—enable separate quantification of adult and paediatric CI service response.

### 2.3. Definitions of Dispensary Indicators

Form No. 12 reports three distinct indicators for each ICD-10 code that are easily confused but represent fundamentally different epidemiological constructs. We use these indicators throughout under the following definitions:

- **Registered annual morbidity** (**reg**): all cases for the diagnosis registered during the reporting year, including first-time and repeat presentations of known cases. This indicator measures healthcare utilisation volume; it is not equivalent to point prevalence in the WHO/GBD epidemiological sense.
- **First-time-in-life registration** (**first**): cases registered with the diagnosis for the first time in the patient’s life. This is the closest dispensary proxy for incidence but is fundamentally an administrative indicator of first contact with the formal healthcare system, not population incidence.
- **Active dispensary observation** (**obs**): patients enrolled in active dispensary surveillance for the diagnosis at year-end. This is closest to treated point-prevalence among care-seeking patients but is selective for those with conditions warranting active long-term follow-up.

Throughout the manuscript, we therefore use the terms ‘registered annual morbidity’, ‘first-time-registered cases’, and ‘active dispensary observation’ rather than ‘prevalence’ and ‘incidence’. We treat these dispensary indicators as healthcare-utilisation measures rather than as population-based epidemiological measures.

### 2.4. Population Denominators

Annual mid-year population estimates by region were obtained from the Bureau of National Statistics of the Republic of Kazakhstan. Adult (≥18 years) and child (0–17 years) population denominators were derived from the same source. The total population covered ranged from 18,630,920 (2020) to 20,283,399 (2025); the adult population from 12,522,759 to 13,380,907.

### 2.5. Statistical Analysis

Crude rates were calculated per 100,000 of the corresponding population, with Wilson score 95% confidence intervals. CI procedure rates were calculated separately per million adults (for adult CI) and per million children (for child CI)—we use age-specific denominators throughout the CI analysis to avoid the artefact of comparing adult-burden indicators against total CI volume that includes paediatric procedures.

Trend significance over time was assessed using three complementary approaches: (i) compound annual growth rate (CAGR); (ii) linear regression; and (iii) the Mann–Kendall non-parametric trend test [16]. Because 20 indicators were tested in parallel, Benjamini–Hochberg false-discovery-rate (FDR) correction was applied across the family of Mann–Kendall *p*-values. The full family of 20 indicators subjected to Benjamini–Hochberg correction, together with their raw and adjusted *p*-values, is presented in Supplementary Table S1. Sex and age contrasts in rates across years were tested with the Wilcoxon signed-rank test on paired annual observations (*n* = 6 pairs). Bivariate regional associations were quantified using Spearman rank correlation. K-means clustering (k = 3) was retained as an exploratory descriptive analysis of regional profiles and was interpreted cautiously given the small number of regions. Three sensitivity analyses were conducted: (a) excluding regions affected by the 2022 administrative reform, (b) excluding Almaty city and Astana city as outliers, and (c) progressively removing the most influential observations from regional Spearman correlations. Data management and core statistical analyses were conducted in Stata version 16.0; exploratory clustering and selected visualisations were produced in Python 3.12. Statistical significance was set at α = 0.05 (two-sided).

### 2.6. Benchmarking Against GBD 2021

To place the Kazakhstani administrative layers in epidemiological context, we benchmarked findings against the GBD-2021 Kazakhstan-specific age-standardised prevalence rate of 17,212 per 100,000 (≥20 dB HL in better ear) and YLD rate of 512 per 100,000 reported by Bukenova et al. (2026) [12]. Global GBD-2021 ASPR (18,070 per 100,000) and regional ASPR estimates (Western Europe 11,917; Eastern Europe 16,268; Central Asia 16,010; East Asia 21,884; South Asia 19,034 per 100,000) from Shen et al. (2025) [1] provided regional context. CI service-response benchmarking used contemporary national registries from Italy [17], Germany [18], and Japan [19], with adult-specific CI rates per million adults computed where possible. We are explicit throughout that GBD modelled prevalence and Kazakhstani administrative indicators measure different constructs and are not directly comparable as competing prevalence estimates; GBD is used as a reference frame to interpret administrative findings, not as a benchmark the administrative system is expected to match.

## 3. Results

We present results in four parts: dispensary registration as the principal administrative epidemiological source (Section 3.1); disability certification as a severe administrative endpoint, with sex, age, and regional disaggregation (Section 3.2, Section 3.3, Section 3.4 and Section 3.5); cochlear implantation as the specialised service response with explicit separation of adult and paediatric uptake (Section 3.6); and finally a comparison of these administrative layers with the GBD-2021 modelled audiometric estimate as an external reference frame (Section 3.7).

### 3.1. Dispensary Registration of Ear and Hearing-Loss Diagnoses

Among adults, the registered annual morbidity for the broad H60–H95 category (all ear and mastoid diseases) grew from 231,209 in 2020 to 278,897 in 2025 (CAGR +3.8%; Mann–Kendall τ = +1.00; FDR-adjusted *p* = 0.016; Table 1). First-time-in-life H60–H95 registrations rose more steeply, from 126,513 to 167,674 (CAGR +5.8%; FDR *p* = 0.016), corresponding to a 2025 first-time registration rate of 1253 per 100,000 adults. By contrast, the number of adults under active dispensary observation for H60–H95 at year-end declined modestly from 21,840 to 19,881 (CAGR −1.9%; FDR *p* = 0.18, not significant). This pattern—rising first-time registrations alongside stable or declining active observation—is compatible with several non-exclusive mechanisms: increased episodic healthcare contact under expanded Compulsory Social Health Insurance coverage, post-pandemic recovery of outpatient services, and secular changes in coding or reporting completeness. The aggregate administrative data used here cannot discriminate between these mechanisms; the trend should not be read as evidence of a rising underlying population incidence of adult ear disease.

For H65–H66 (chronic otitis media—a key potentially preventable contributor to conductive hearing loss), adult first-time registration remained broadly stable across the study period (16,651 → 17,882; FDR *p* = 0.32), with values around 130–160 per 100,000 adults annually. The substantial decline in active observation (6871 → 2245; CAGR −20.0%; FDR *p* = 0.016) should be interpreted cautiously. It may reflect improved clinical resolution, changes in follow-up practice, or administrative reclassification of chronic otitis cases out of active surveillance pools; the aggregate data cannot distinguish these explanations.

**Figure 1:**
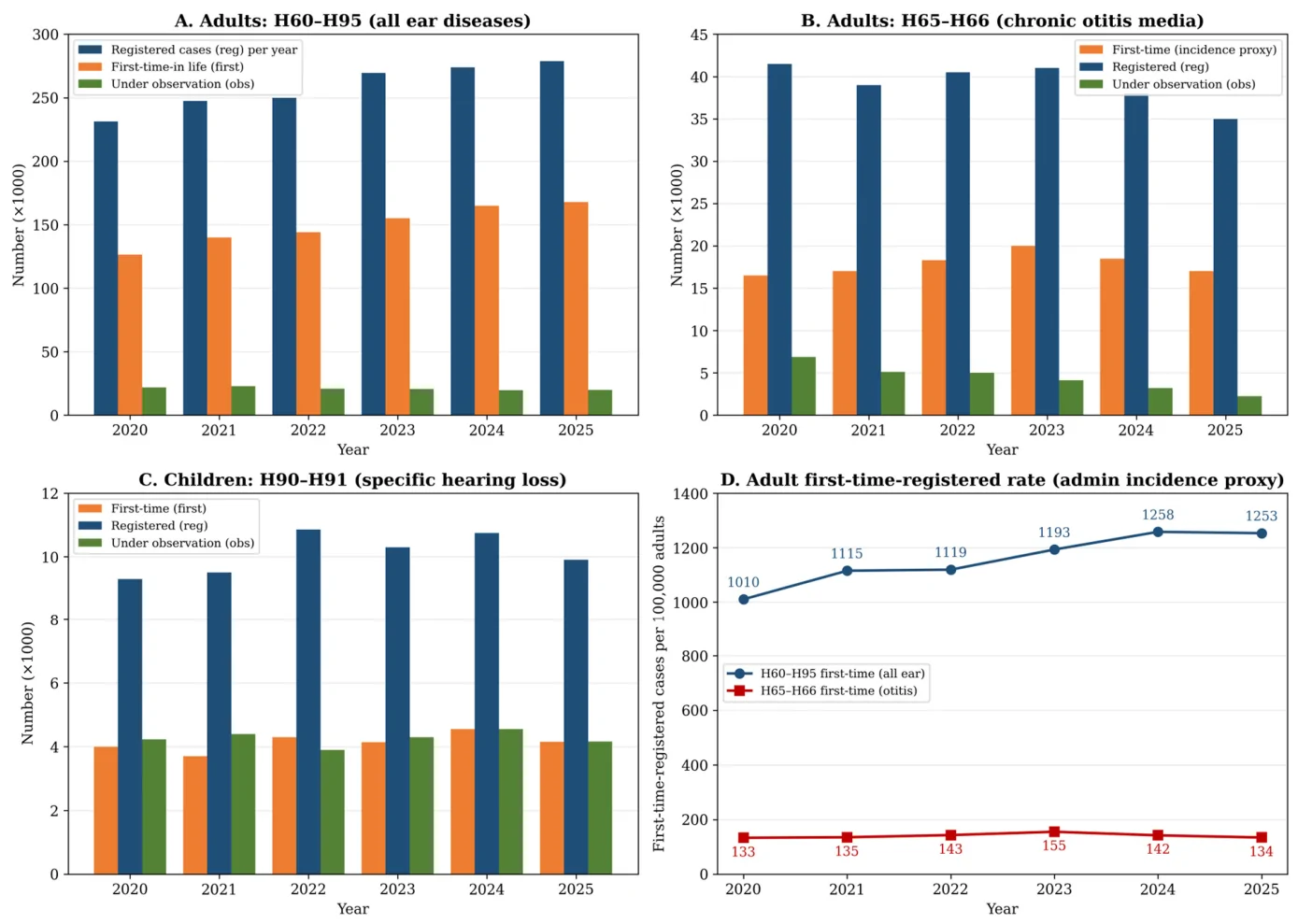
Dispensary registration of ear diseases and hearing loss in Kazakhstan, 2020–2025. (**A**) adults with H60–H95 (all ear and mastoid disease); (**B**) adults with H65–H66 (chronic otitis media); (**C**) children with H90–H91 (specific hearing loss diagnoses); (**D**) adult first-time-registered rate per 100,000 adults—H60–H95 versus H65–H66.

For children, H90–H91 (specific hearing loss diagnoses—conductive, sensorineural, and mixed) showed no significant trend across the study period (first-time: 3990 → 4148, FDR *p* = 0.53; active observation: 4226 → 4159, FDR *p* = 1.00). This stability is compatible with a mature universal newborn hearing screening (UNHS) pathway, operational nationally since 2010 [20], although the aggregate data do not allow us to evaluate screening coverage, diagnostic delay, or follow-up completeness at the individual level. Figure 1 displays the dispensary indicator trajectories.

### 3.2. Disability Certification: The Severe Administrative Endpoint

The disability registry identifies the severe certified end of the hearing-loss administrative spectrum, comprising adults with grade IV bilateral hearing loss not amenable to hearing-aid correction (or complete deafness) and children with grade III bilateral hearing loss or worse. Between 2020 and 2025, the total number of persons registered with hearing impairment-related disability grew from 29,743 to 31,560 (CAGR +1.2%; FDR *p* = 0.016; Table 2). Adults consistently comprised 84% of all registrants. The crude registered disability rate ranged narrowly from 159.6 (2020) to 155.6 (2025) per 100,000, and the adult-specific registered disability rate stabilised at approximately 198 per 100,000 from 2022 onwards (FDR *p* = 0.74, not significant). The dynamics of disability registration are therefore much flatter than those of dispensary morbidity, consistent with the registry capturing only the severe certified subset.

### 3.3. Sex Disparities in Disability Registration

Males consistently demonstrated higher registered disability rates than females across all six study years. Male-specific rates ranged from 173.2 (2020) to 164.8 (2025) per 100,000 men; female rates ranged from 146.9 to 146.8 per 100,000 women. The male-to-female rate ratio narrowed from 1.18 in 2020 to 1.12 in 2025, but the difference remained statistically significant (Wilcoxon signed-rank test: W = 0, *p* = 0.031). The persistent male predominance is consistent with global GBD-2021 findings (male ASPR 19,197 vs female 16,979 per 100,000) [1] and with the established association of occupational and recreational noise exposure with male-predominant industries [21,22].

### 3.4. Age Disparities in Disability Registration

Adult-specific disability rates substantially exceeded child-specific rates across all years (adults 197.7–200.6 vs children 73.0–85.0 per 100,000; ratio 2.6–2.7; Wilcoxon W = 0, *p* = 0.031). The State Register reports only the binary adult/child classification, so the full age-cumulative gradient cannot be quantified from administrative records. GBD-2021 Kazakhstan-specific estimates indicate the highest adult prevalence in the 70–79 and 80+ age strata [12], suggesting that the bulk of registered adult disability concerns older age groups.

### 3.5. Regional Variation in Disability Registration

**Figure 2:**
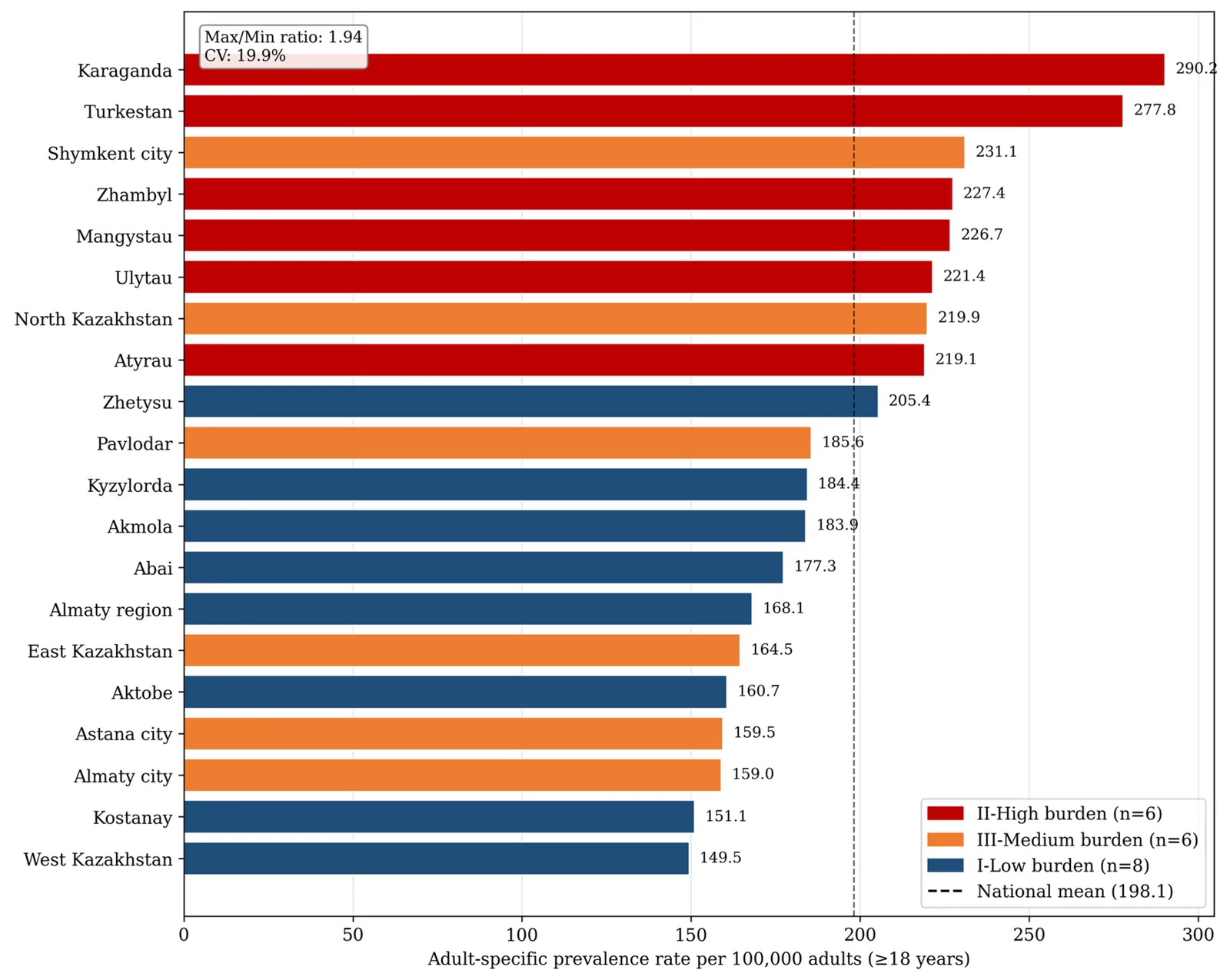
Regional disparities in registered adult hearing disability, Kazakhstan 2025. Bars sorted ascending; colours indicate cluster membership from k-means analysis (k = 3) on standardised regional rates.

Substantial interregional variation in adult-specific disability rates was observed in 2025 (Table 3, Figure 2). The mean regional adult rate was 198.1 per 100,000 adults (SD 39.4; median 185.0; CV 19.9%). The highest adult rate was in Karaganda oblast (290.2 per 100,000), followed by Turkestan (277.8), Shymkent city (231.1), Zhambyl (227.4), Mangystau (226.7), and Ulytau (221.4); the lowest was West Kazakhstan (149.5), yielding a max-to-min ratio of 1.94.

Exploratory k-means clustering (k = 3, on z-standardised total, adult, and child registered disability rates) identified three regional profiles. Cluster II (high burden, *n* = 6 regions)—Karaganda, Turkestan, Zhambyl, Mangystau, Ulytau, and Atyrau—had a mean adult rate of 243.8/100,000, approximately 23% above the national mean. Cluster III (medium burden, *n* = 6 regions) included the major cities (Shymkent, Astana, Almaty) plus North Kazakhstan, Pavlodar, and East Kazakhstan (mean adult rate 186.6). Cluster I (low burden, *n* = 8 regions) comprised mainly western and northern oblasts (mean rate 172.5). The high-burden cluster includes several industrial regions (Karaganda mining basin; Mangystau and Atyrau oil-and-gas; Ulytau coal-mining) and southern regions with large rural populations (Turkestan, Zhambyl). Silhouette score (0.39) and bootstrap stability (74%) indicate only moderate separation. The three-cluster solution should therefore be read strictly as an exploratory descriptive summary of the 2025 regional profile, not as a validated classification of regional risk. Cluster labels are used in the text and figures only for exposition; individual regions should not be interpreted as belonging to intrinsic “burden categories”.

### 3.6. Cochlear Implantation Service Response

**Figure 3:**
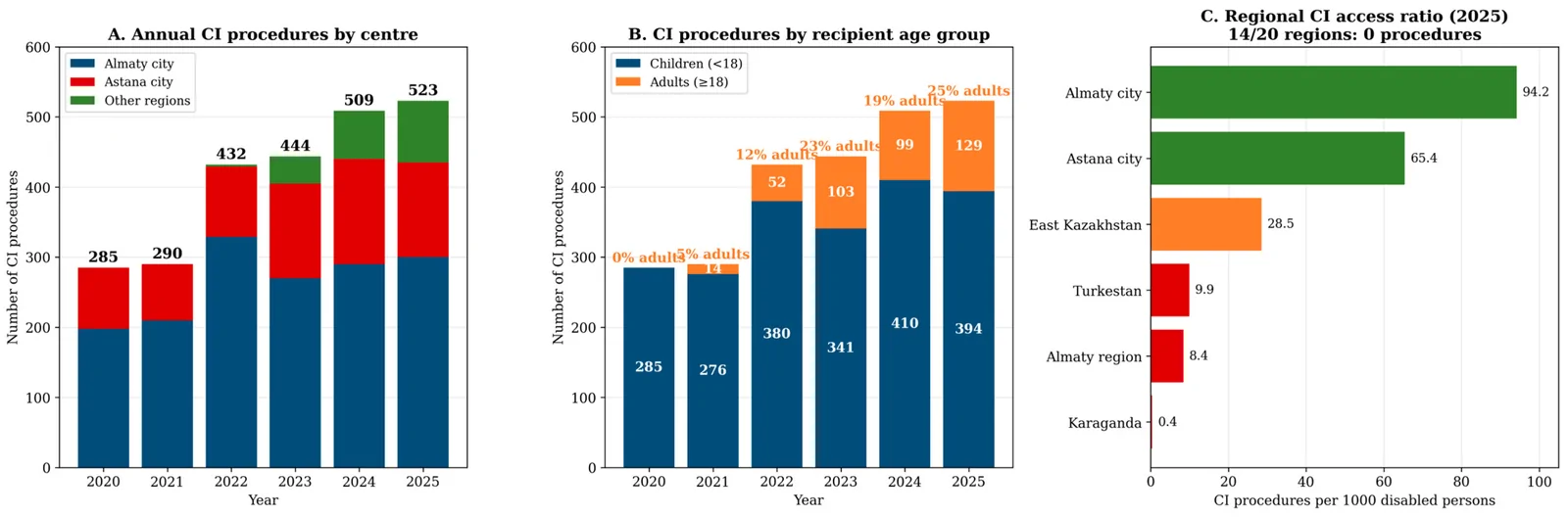
Cochlear implantation in Kazakhstan, 2020–2025: (**A**) annual volume by performing centre; (**B**) adult versus child recipient split with adult share percentage; (**C**) regional access ratio per 1000 registered HI disabled persons (2025). Fourteen of 20 regions performed no CI procedures in 2025.

Cochlear implantation in Kazakhstan expanded substantially across the study period (Table 4, Figure 3). Total annual volume grew from 285 in 2020 to 523 in 2025 (CAGR +12.9%; FDR *p* = 0.016). For adult-focused analysis, the relevant indicator is adult CI procedures, not total CI: the adult CI count grew from 0 in 2020 to 129 in 2025, with new adult procedures resuming in 2021 (*n* = 14) following a two-year pause in 2019–2020, and a steady upward trajectory thereafter. Although cochlear implantation was introduced in Kazakhstan in 2007, the earliest year with age-stratified administrative records is 2012. Between 2012 and 2018, adult CI activity was intermittent (annual volumes: 125, 140, 24, 11, 63, 29, and 33; cumulative 425 procedures), followed by a complete pause in 2019–2020 (0 procedures in each year, consistent with Table 4). The cumulative 2012–2019 all-age CI volume was 1486 procedures. Consistent year-on-year scaling of adult CI began in 2021 (*n* = 14) and continued through 2025 (*n* = 129; FDR *p* = 0.037 for trend). By 2025, adults accounted for 24.7% of all CI procedures. However, consistent year-on-year scaling of adult CI began only in 2021 (FDR *p* = 0.037 for trend). By 2025, adults accounted for 24.7% of all CI procedures. Paediatric CI also grew, from 285 to 394 (CAGR +6.7%), but the trend did not survive FDR correction (FDR *p* = 0.18).

To assess service response relative to identified need, we calculated age-specific CI ratios. The adult CI service-response rate reached 9.6 procedures per million adults in 2025, or 4.86 procedures per 1000 registered adult disabled persons. The paediatric CI rate was substantially higher: 57.1 per million children, or 78.2 per 1000 registered disabled children. This adult-paediatric asymmetry reflects both the historical paediatric focus of Kazakhstan’s CI programme (built around UNHS-detected congenital cases) and the more recent introduction of adult CI indications.

Geographic concentration of CI services remains pronounced. In 2025, Almaty city accounted for 300 of 523 procedures (57.4%) and Astana city for 135 (25.8%), together representing 83.2% of all cochlear implantations. New CI activity emerged in Turkestan (40 procedures, of which 27 were in adults), East Kazakhstan (30, all paediatric), and Almaty region (17, of which 16 were in adults), but 14 of 20 regions performed zero CI procedures in 2025. Because the register is analysed by performing facility, these figures describe the geography of service provision rather than the residence of every patient. Spearman correlation between regional CI procedure count and total registered HI disability count was statistically significant in 2025 (ρ = +0.56, *p* = 0.010, *n* = 20), reflecting recent CI activity in three high-burden regions. The correlation is sensitive to the largest centre: dropping Almaty city reduces it to ρ = 0.48 (*p* = 0.040), and dropping both Almaty and Astana reduces it to ρ = 0.40 (*p* = 0.10). We therefore interpret this correlation as suggestive of early need-oriented expansion rather than evidence of a system-wide allocation shift.

### 3.7. Administrative Layers in the Reference Frame of the GBD 2021 Modelled Estimate

**Figure 4:**
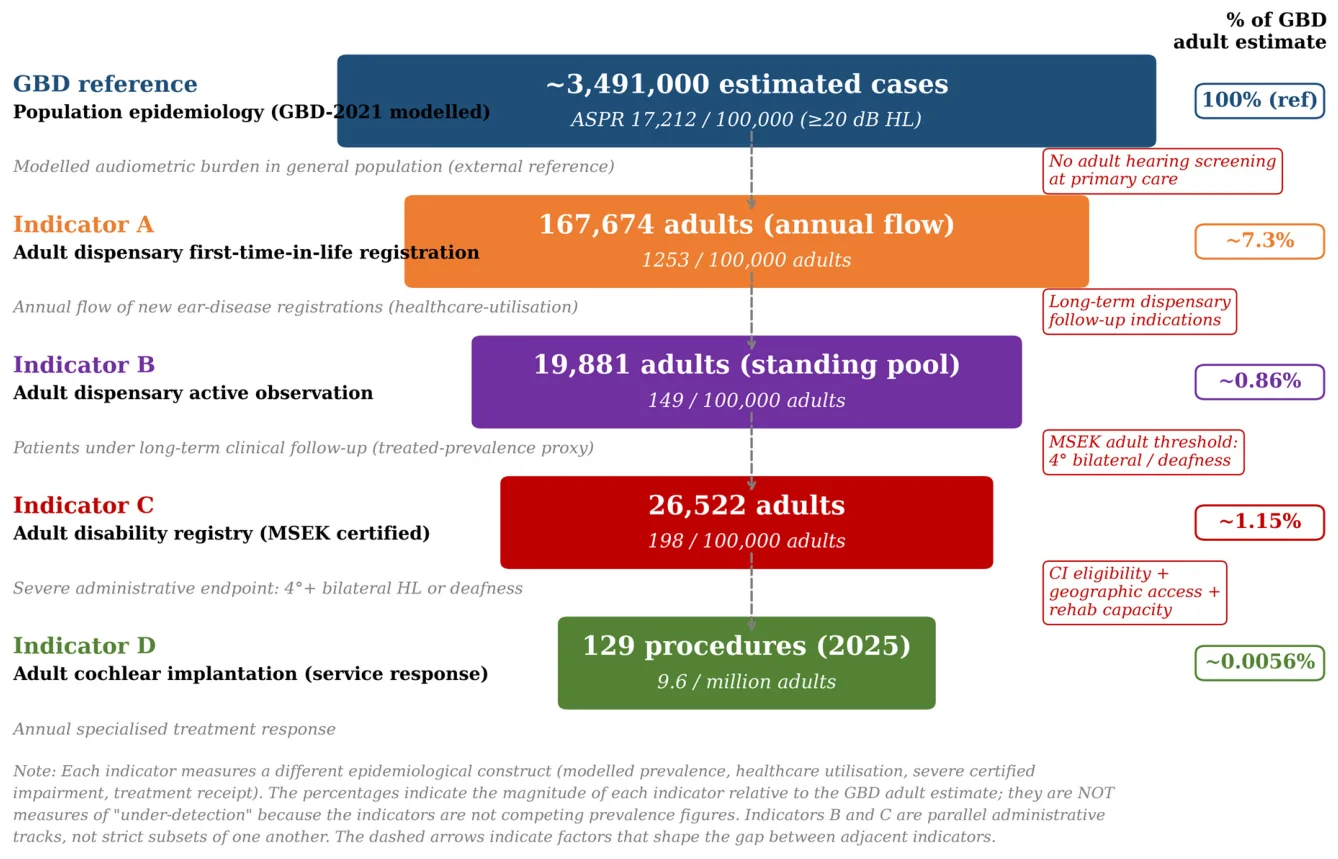
Conceptual cascade of hearing care in Kazakhstan’s administrative ecosystem, shown with 2025 quantitative values against the GBD-2021 modelled audiometric reference. Each successive layer—modelled audiometric burden, dispensary first-time registration, dispensary active observation, disability certification, and adult cochlear implantation—measures a distinct epidemiological construct rather than a subset of the layer above. Side annotations identify the health-system mechanisms shaping the width difference between adjacent layers (absence of adult hearing screening at primary care level; long-term dispensary follow-up indications; MSEK adult severity threshold; CI eligibility, geographic access, and rehabilitation capacity). Percentages are scale markers of each layer’s magnitude relative to the modelled adult population burden, not estimates of under-detection.

Figure 4 and Table 5 together place the four administrative layers in relation to the GBD-2021 Kazakhstan-specific modelled audiometric estimate. The GBD-2021 age-standardised prevalence rate of 17,212 per 100,000 [12] corresponds to approximately 3.49 million estimated cases at any audiometric severity (≥20 dB HL) in the total 2025 Kazakhstani population (20,283,399 × 17,212/100,000), and approximately 2.30 million estimated cases in the adult (≥18 years) population (13,380,907 × 17,212/100,000). Because the administrative indicators reported in this section are adult-specific, we express the relation of each indicator as a percentage of the adult-only GBD reference (2.30 million estimated adult cases). Percentages are therefore scale markers of each administrative layer’s magnitude relative to the modelled adult population burden, not estimates of under-detection. Of this modelled population pool, the total adult H60–H95 registered annual morbidity (all ear-disease registrations, including first-time and repeat presentations) corresponds to approximately 12.1% as the broadest administrative visibility of ear and hearing-related healthcare contact; within this, the adult first-time-in-life registration captures approximately 7.3% as the annual incident flow into the formal healthcare system; the adult active dispensary observation captures approximately 0.86% as a long-term clinical care pool; the adult disability registry captures approximately 1.15% as severely impaired persons certified by MSEK; and adult cochlear implantation reaches approximately 0.0056% as the annual specialised treatment response. The percentages scale markers, not estimates of under-detection. They help locate each administrative indicator against the magnitude of the modelled population burden, while preserving the fact that the indicators measure different constructs.

**Figure 5:**
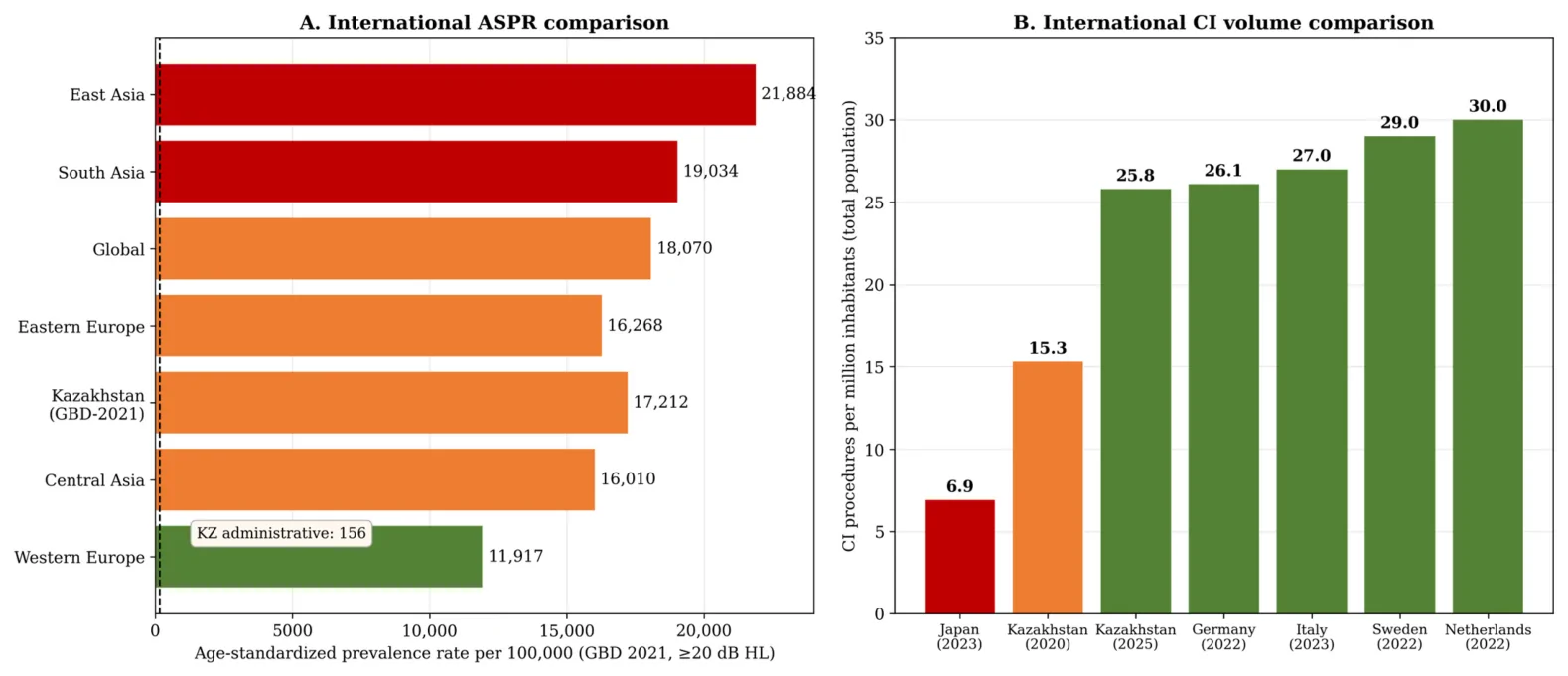
International benchmarking: (**A**) Kazakhstan administrative crude prevalence and adult disability registration in relation to GBD-2021 ASPR for Kazakhstan and selected world regions; (**B**) cochlear implantation volume per million inhabitants—Kazakhstan compared with Italy, Germany, Japan, Sweden, and the Netherlands.

This reference-frame approach also clarifies what international CI registry comparisons can and cannot tell us about Kazakhstan’s adult CI service response. Table 6 and Figure 5 contrast adult CI rates per million adults across countries with publicly available registry data. Kazakhstan’s adult CI rate of 9.6 per million adults in 2025 appears lower than approximate adult-CI rates reported or derivable from Italian and German registry data, and broadly in the range suggested by Japanese registry distributions. However, the international adult-subset values are approximate and should not be treated as exact like-for-like comparisons. Kazakhstan’s total CI rate per million population (25.8) is similar to several Western European aggregate rates, but this aggregate is dominated by paediatric procedures and is not informative for adult-burden service planning.

## 4. Discussion

This study makes three contributions to the evidence on hearing health in Kazakhstan. First, it integrates three independent administrative sources rather than relying on a single register. Second, it separates dispensary indicators that have often been conflated in routine reporting—registered annual morbidity, first-time-in-life registration, and active dispensary observation—and interprets them as healthcare-utilisation measures rather than as prevalence or incidence. Third, it uses age-stratified cochlear implantation data to distinguish adult and paediatric service response for the first time in a national analysis.

### 4.1. What the Dispensary Layer Tells Us

Among adults, registered annual H60–H95 morbidity (CAGR +3.8%) and first-time-in-life registration (CAGR +5.8%) increased, while active observation was stable or declining. The most cautious interpretation is increased episodic contact with the formal health system for ear and mastoid disease, without a matching increase in long-term follow-up. Possible explanations include improved access under the Compulsory Social Health Insurance programme, recovery of outpatient services after the pandemic period, and greater awareness or care-seeking for ear-related complaints. These data should not be read as evidence that true population incidence of adult ear disease is rising at 5.8% per year; the first-time indicator measures first recorded contact with the system.

The H65–H66 chronic otitis findings suggest a relatively stable flow of newly registered adult cases, with a marked decline in active observation. This could reflect improved clinical resolution, changes in follow-up rules, or administrative reclassification; the aggregate data cannot resolve the mechanism. Paediatric H90–H91 registration was stable across all three indicators, a pattern consistent with a mature newborn hearing screening pathway, but not sufficient to evaluate individual-level coverage or outcomes.

Children’s H90–H91 dispensary registration was stable across all three indicators. This stability is compatible with a mature universal newborn hearing screening pathway [11], but the aggregate registration counts alone cannot confirm that the UNHS pipeline is functioning as intended. Evaluation of screening coverage, refer-rate, diagnostic delay to confirmatory audiometry, and lost-to-follow-up rates would require individual-level linkage of screening, diagnostic, and dispensary records and remains a priority for future work.

### 4.2. Disability Certification as a Severe Administrative Endpoint

Disability registration in Kazakhstan operates under the medico-social expert evaluation (MSEK) framework, which requires both an audiometric criterion and a functional limitation criterion. For adults, the threshold is grade IV bilateral hearing loss not correctable with hearing aids, or complete deafness; for children, grade III bilateral hearing loss is sufficient. This asymmetry reflects the Kazakhstani system’s underlying logic—adult disability is conceptually anchored in loss of work capacity, with the implicit assumption that hearing aids restore sufficient function for adults to remain economically active in most occupations. The result is that disability registration is a highly selective indicator: it captures the severe end of the hearing-loss spectrum in adults and a slightly broader range in children, and it should be interpreted as such.

In our data, the adult registered disability rate was essentially flat at approximately 198 per 100,000 adults across 2020–2025, despite the 5.8 per-year rise in adult H60–H95 first-time registration. This decoupling is expected: most first-time ear-disease registrations do not have severe enough hearing loss to meet adult MSEK criteria. The disability count does, however, reveal substantial regional variation (CV 19.9%; max-to-min ratio 1.94). The high-burden cluster—Karaganda, Turkestan, Zhambyl, Mangystau, Ulytau, and Atyrau—includes industrial regions with plausible occupational noise risks [23] and southern regions with historically limited specialist access. Because individual exposure and residence data are unavailable, these contextual explanations should be treated as hypotheses rather than causal findings.

### 4.3. Cochlear Implantation as a Service Response

Reading the CI data through an adult-focused lens reveals two findings that are obscured if total CI volumes are reported in isolation. First, adult cochlear implantation in Kazakhstan was re-established as a sustained programme during this study period, following intermittent activity since 2012 and a two-year pause in 2019–2020. National records show 125 adult procedures in 2012 and intermittent activity through 2018, after which adult CI paused and then resumed in 2021 (*n* = 14), scaling steadily to 129 procedures in 2025. By the end of the period, adults represented one in four CI recipients. This trajectory reflects a transition from sporadic adult CI activity to a consistent, year-on-year adult CI service alongside the long-standing paediatric programme [10,24]. Second, the adult CI service-response rate—9.6 procedures per million adults in 2025, or 4.86 per 1000 registered adult disabled persons —remains below approximate rates from established European programmes [17,18]. The total CI rate per million population (25.8) appears comparable to Western Europe, but this aggregate is dominated by paediatric procedures and should not be used for adult-burden service planning.

Geographic concentration is a defining feature of the CI service response. With 83% of procedures performed in Almaty and Astana and 14 of 20 regions performing none in 2025, the spatial distribution of CI reflects the location of two specialised centres. This does not necessarily mean that patients from other regions are not being treated there; it means that the service delivery platform is concentrated. The 2025 Spearman correlation between regional CI volume and disability count (ρ = +0.56, *p* = 0.010) suggests early expansion in several high-burden regions, but the association is fragile to the removal of the two largest cities. We do not interpret concentration as a programme failure. Replicating CI surgical capacity in 17 additional regions is neither feasible nor desirable in the short term, given the technical complexity and the need for multidisciplinary teams (otosurgeon, audiologist, speech-language pathologist, and neurolinguist) [25]. A more realistic model is centralised surgery combined with regional audiology and rehabilitation hubs for pre-operative assessment and post-implant programming.

Given the geographic concentration of CI surgical capacity in Almaty and Astana and the need for accessible post-implant rehabilitation across all 20 regions, digital and AI-supported tools warrant explicit consideration in future service design. Chat-based mobile auditory training has shown feasible use, high adherence, and measurable gains in word- and sentence-level perception in adult hearing-aid users [26], suggesting a scalable model for remote rehabilitation adherence monitoring and patient education. In parallel, emerging AI-enabled innovations in cochlear implant technology—spanning candidacy prediction, automated fitting, and outcome monitoring [27]—may reduce the specialist-time barrier that currently constrains adult CI scale-up. Both directions align with the regional hub-and-spoke architecture proposed above and with the priority we identify for a longitudinal outcome registry.

### 4.4. Reading Administrative Indicators Against the GBD Reference Frame

The framing developed in Section 3.7 makes one point essential for health-system planning: the GBD-2021 modelled estimate of approximately 17,212 per 100,000 [12] and the administrative indicators in this study are not competing prevalence estimates. The GBD estimate models the audiometric burden in the general population—what a national audiometric survey might find. The dispensary first-time indicator measures annual first recorded contact with the health system for a broad ear-disease category; active observation measures a standing pool under long-term clinical follow-up; disability certification measures severe impairment formally recognised for social support; and the CI register measures a specialised surgical rehabilitation response. The disability registry and dispensary observation pool are parallel administrative systems with different inclusion logics, not strict subsets of one another.

This distinction matters in practice. A simple headline such as a “99-fold gap” between disability certification and GBD ASPR is technically possible but not very informative, because it compares different constructs. More useful is to ask where the service bottlenecks appear. The difference between the modelled population estimate and dispensary registration is shaped by care-seeking, access to primary care, and the absence of organised adult hearing screening. The difference between first-time registration and active observation is shaped by clinical indications and follow-up rules. The difference between administrative hearing indicators and disability certification reflects MSEK severity thresholds. The difference between disability certification and CI receipt reflects CI indications, geographic access, and rehabilitation capacity. Naming the indicators precisely is therefore not a semantic issue; it changes the policy interpretation.

On Kazakhstan’s place in the international GBD landscape, Shen et al. (2025) report that the GBD-2021 ASPR varies from 11,917 per 100,000 in Western Europe to 21,884 per 100,000 in East Asia [1]. Central Asia (which includes Kazakhstan) has an estimated regional ASPR of 16,010, slightly below the global mean (18,070). The Kazakhstan-specific estimate (17,212) places the country in the upper portion of the Central Asian distribution, consistent with its industrial economy and ongoing demographic ageing. Notably, the GBD pattern across world regions shows a generally inverse relationship between sociodemographic index and ASPR, with East Asia as a striking outlier where both ASPR and YLD rate rise with SDI [1]. Kazakhstan’s situation has parallels with this East Asian pattern: industrial expansion combined with accelerating ageing in a context of incomplete adult hearing surveillance.

### 4.5. International Relevance of the Administrative Cascade Framework

Prior national analyses of hearing health have typically drawn on a single register—a cochlear implant registry, a disability registry, or an audiometric survey—and interpreted the resulting counts as if they answered the same question. The framework applied here treats dispensary registration, disability certification, and cochlear implantation as three parallel administrative layers, each capturing a different construct (healthcare contact, severe certified impairment, specialised treatment response) and each interpretable only against a modelled audiometric reference such as GBD. This ‘cascade of hearing care’ logic is directly transferable to other low- and middle-income countries that operate parallel Semashko-derived or ICD-based administrative reporting systems alongside disability and treatment registries. In such settings, applying the framework requires only that each register be interpreted under its own operational definition and benchmarked against a modelled population estimate rather than against the other registers. The framework does not require a national audiometric survey to yield actionable planning signals about where along the cascade the health system loses visibility of unmet need.

### 4.6. The Adult Hearing-Screening Gap and Recent Regulatory Change

Kazakhstan operates a universal newborn hearing screening programme that has reached comprehensive coverage [20], and our data are consistent with mature paediatric screening. No equivalent programme exists for adults at primary care level. As Bukenova et al. (2024) document, the standard adult preventive examination form (form 055/U) includes screening for cardiovascular disease, diabetes, vision, and oncology, but contains no hearing-related questions or examinations [28]. The HHIA-S questionnaire, validated in Astana with sensitivity 70.2% and specificity 94.1% (AUC 0.82) [12], provides a feasible, low-cost screening tool for routine adult preventive examinations.

Order No. 92 of the Minister for the first time extends state-funded hearing aid provision to non-disabled adults with hearing loss [13], creating a regulatory pathway for population-level intervention beyond the disability framework. This is a meaningful policy shift, but it does not by itself implement the screening pathway needed to identify candidates. Operationalising adult hearing screening at primary care level remains a priority for health-system development.

### 4.7. Strengths and Limitations

Principal strengths include national coverage of all 20 regions; integration of three complementary administrative data sources (114 region-year observations × 111 variables); explicit disambiguation of dispensary indicator types (reg/first/obs) and ICD-10 subdivisions (H60–H95, H65–H66, H90–H91); newly available adult/child age stratification of CI procedures; and explicit framing of GBD-2021 as an external reference frame rather than a competing prevalence estimate.

Key limitations include: First, all analyses are based on aggregated administrative data reflecting healthcare-utilising or formally certified populations, not population-based audiometric surveys. Second, dispensary indicators (reg/first/obs) are healthcare-utilisation measures, not direct epidemiological prevalence or incidence; they are influenced by access to care, reporting practices, and clinical follow-up rules. Third, H60–H95 is a broad ear-and-mastoid disease category and should not be treated as a hearing-loss-specific comparator to GBD estimates. Fourth, hearing-loss severity, aetiology, audiometric thresholds, hearing-aid uptake, and rehabilitation outcomes are not captured in the administrative records. Fifth, the dispensary form reports H90–H91 separately only for children; adult hearing-loss-specific cases are aggregated within H60–H95, which limits direct adult comparison with GBD hearing-loss estimates. Sixth, individual-level age data within the adult category (≥18 years) are unavailable; direct age standardisation by 5-year age groups could not be performed. Seventh, the limited number of annual observations (*n* = 6) constrains statistical power for trend detection. Eighth, the 2022 administrative reform affects time-series comparability for source regions, although sensitivity analyses supported the main conclusions. Ninth, cochlear implantation data are analysed by region of performing facility, not necessarily by patient residence; regional CI concentration therefore reflects service location and should not be interpreted as the geographic origin of all recipients. Tenth, individual-level data on socioeconomic status, occupational exposure, ototoxic medication history, and clinical outcomes after CI were unavailable. These limitations mean that the study cannot replace a population-based audiometric survey; rather, it shows why such a survey would be valuable.

## 5. Conclusions

Kazakhstan’s hearing-health administrative ecosystem is best interpreted as a set of parallel but related layers: dispensary registration, disability certification, and cochlear implantation. Each measures a different construct and is most useful when read alongside, rather than directly against, the GBD-2021 modelled estimate. Adult H60–H95 first-time-in-life registration is rising at approximately 5.8% per year, while active observation is stable or declining—consistent with increasing episodic healthcare contact without proportional growth in long-term follow-up infrastructure. Disability certification is essentially flat at about 198 per 100,000 adults, capturing only the most severe cases under the grade IV-bilateral threshold. Cochlear implantation has expanded 1.8-fold to 25.8 per million population, with adult CI re-established as a sustained programme (129 procedures in 2025), building on intermittent activity since 2012 and a two-year pause in 2019–2020. The adult-only rate of 9.6 per million adults remains below approximate European adult-CI rates, and geographic concentration in two cities remains pronounced.

The differences between administrative indicators and the GBD-2021 modelled estimate should not be reduced to a single under-detection figure. They are better understood as signals about where the health system records, follows, certifies, and treats hearing loss. The largest gap is between the modelled audiometric burden and the adult first-time dispensary registration layer, which points to the absence of a systematic adult hearing-screening pathway at primary care level. A second important gap lies between adult registered disability and adult CI receipt, highlighting the need to strengthen adult referral, assessment, and rehabilitation pathways.

The administrative patterns documented here support consideration of six policy directions rather than establishing them as proven intervention priorities: (1) operationalising adult hearing screening at primary care level using validated brief instruments (HHIA-S, whisper test, mobile audiometry), enabled by the regulatory pathway opened by Order No. 92 of 2025 [13]; (2) integrating routine hearing examination into the standard adult preventive examination form (form 055/U); (3) developing regional satellite audiology and rehabilitation hubs to support pre-operative assessment and post-implant programming, with CI surgery consolidated in two or three centres of excellence; (4) targeted occupational hearing surveillance in identified high-burden industrial regions (Karaganda, Pavlodar, Atyrau, Mangystau); (5) establishing a national hearing implant registry analogous to RIPI/RIDIU [14] or DCIR [15] to enable longitudinal outcome monitoring; and (6) progressive alignment of Kazakhstan’s disability classification with the international biopsychosocial framework, without dismantling the social protections currently provided through the labour-capacity model. Each of these directions warrants formal evaluation before implementation at scale.

The administrative cascade framework applied here is transferable to other low- and middle-income countries that maintain parallel disease-notification, disability, and treatment registries alongside modelled epidemiological estimates.

## Figures and Tables

**Table 1:** Dispensary registration of ear and hearing-loss diagnoses, Kazakhstan 2020–2025.

Indicator (Adults Unless Noted)	2020	2025	CAGR	MK τ	MK *p* (FDR)	Sig. (FDR)
H60–H95 registered annual morbidity	231,209	278,897	+3.8%	+1.00	0.016	✓
H60–H95 first-time-in-life	126,513	167,674	+5.8%	+1.00	0.016	✓
H60–H95 active dispensary observation	21,840	19,881	−1.9%	−0.60	0.18	—
H65–H66 first-time (chronic otitis)	16,651	17,882	+1.4%	+0.47	0.32	—
H65–H66 active dispensary observation	6871	2245	−20.0%	−1.00	0.016	✓
H90–H91 first-time (children only)	3990	4148	+0.8%	+0.33	0.53	—
H90–H91 active observation (children only)	4226	4159	−0.3%	+0.07	1.00	—

Note: Indicators defined in Section 2.3. **MK τ** = Mann–Kendall trend statistic. **MK *p* (FDR)** = Mann–Kendall *p*-value after Benjamini–Hochberg false-discovery-rate (FDR) correction, applied across the family of 20 Mann–Kendall tests reported in this study (see Supplementary Table S1 for the full set). **Sig. (FDR)** indicates whether the FDR-corrected *p*-value falls below the significance threshold of 0.05: ✓ = significant after FDR correction; — = not significant after FDR correction.

**Table 2:** Disability registry—hearing impairment cases, Kazakhstan 2020–2025.

Year	Total	Adults	Children	Male	Female	Crude Rate (95% CI)	Adult Rate (95% CI)	% Adults
2020	29,743	24,970	4773	15,642	14,101	159.6 (157.8–161.5)	199.4 (196.9–201.9)	83.9%
2021	30,128	25,242	4886	15,812	14,316	159.6 (157.8–161.4)	200.6 (198.1–203.0)	83.8%
2022	30,464	25,471	4993	15,900	14,564	156.2 (154.5–158.0)	197.7 (195.3–200.1)	83.6%
2023	30,782	25,809	4973	16,031	14,751	155.7 (154.0–157.5)	198.1 (195.7–200.5)	83.8%
2024	31,180	26,152	5028	16,189	14,991	155.6 (153.9–157.4)	198.2 (195.8–200.7)	83.9%
2025	31,560	26,522	5038	16,334	15,226	155.6 (153.9–157.3)	198.2 (195.8–200.6)	84.0%

Note: 95% confidence intervals computed using the Wilson score method for proportions.

**Table 3:** Regional distribution of registered hearing disability, Kazakhstan 2025 (sorted by adult-specific rate).

Region	Total	Adults	Children	Male	Female	Pop. (Adults)	Adult Rate/100 k	Cluster
Karaganda	2578	2377	201	1304	1274	819,141	290.2	II
Turkestan	4030	3409	621	2164	1866	1,227,228	277.8	II
Shymkent city	2174	1724	450	1085	1089	746,120	231.1	III
Zhambyl	2046	1729	317	1050	996	760,289	227.4	II
Mangystau	1304	1064	240	643	661	469,396	226.7	II
Ulytau	362	323	39	203	159	145,863	221.4	II
North Kazakhstan	988	877	111	530	458	398,787	219.9	III
Atyrau	1135	953	182	572	563	434,942	219.1	II
Zhetysu	1068	928	140	571	497	451,883	205.4	I
Pavlodar	1185	1018	167	624	561	548,429	185.6	III
Kyzylorda	1191	941	250	658	533	510,197	184.4	I
Akmola	1177	1040	137	636	541	565,553	183.9	I
Abai	845	739	106	434	411	416,693	177.3	I
Almaty region	2026	1673	353	1071	955	995,199	168.1	I
East Kazakhstan	1053	893	160	511	542	542,701	164.5	III
Aktobe	1217	988	229	632	585	614,937	160.7	I
Astana city	2063	1629	434	1020	1043	1,021,530	159.5	III
Almaty city	3186	2563	623	1581	1605	1,612,183	159.0	III
Kostanay	1075	940	135	579	496	622,095	151.1	I
West Kazakhstan	857	714	143	466	391	477,741	149.5	I

Note: Cluster I = low burden; Cluster II = high burden; Cluster III = medium burden. K-means clustering performed on z-standardised regional rates with k = 3.

**Table 4:** Cochlear implantation service response, Kazakhstan 2020–2025.

Indicator	2020	2021	2022	2023	2024	2025	CAGR/MK *p* (FDR)
Total CI procedures	285	290	432	444	509	523	+12.9%; FDR 0.016 ✓
Adult CI (≥18)	0	14	52	103	99	129	—; FDR 0.037 ✓
Child CI (<18)	285	276	380	341	410	394	+6.7%; FDR 0.18
Adult CI per million adults	0.00	1.11	4.04	7.91	7.50	9.64	—
Child CI per million children	46.7	43.9	57.4	50.6	59.9	57.1	+4.1%
Adult CI per 1000 adult disabled	0.00	0.55	2.04	3.99	3.79	4.86	—
Child CI per 1000 children disabled	59.7	56.5	76.1	68.6	81.5	78.2	+5.5%

Source: Electronic Register of Inpatient Patients. Adult CI rates use adult-only denominators (population and disability registry). **CAGR** = compound annual growth rate over 2020–2025. **CAGR/MK *p* (FDR)** column reports the CAGR followed by the Mann–Kendall *p*-value after Benjamini–Hochberg false-discovery-rate (FDR) correction across the family of 20 Mann–Kendall tests reported in this study (see Supplementary Table S1). ✓ indicates the FDR-corrected *p*-value falls below the significance threshold of 0.05; “—” in the CAGR position denotes an undefined CAGR (baseline value = 0).

**Table 5:** Kazakhstan’s hearing-health administrative layers in 2025, with reference to the GBD-2021 modelled estimate.

Administrative Layer	Indicator	Cases/Rate 2025	Relation to GBD Adult Estimate	Interpretation
1. Population (modelled)	GBD-2021 ASPR Kazakhstan	17,212/100,000 (≈3.49 million est. cases)	100% (reference)	Modelled audiometric burden ≥20 dB HL
2. Healthcare utilisation (overall)	Adult H60–H95 registered annual morbidity	278,897 (2084.6/100,000 adults)	~12.1% of GBD adult estimate	All adult ear-disease cases registered during the reporting year (includes first-time and repeat presentations)
2a. Healthcare utilisation (incident flow)	Adult H60–H95 first-time-in-life registration	167,674 (1253/100,000 adults)	~7.3% of GBD adult estimate	Annual flow of new ear-disease registrations
2b. Active clinical care	Adult H60–H95 active dispensary observation	19,881 (149/100,000 adults)	~0.86% of GBD adult estimate	Persons under long-term clinical follow-up
3. Severe administrative endpoint	Adult disability registry (grade IV+ HL)	26,522 (198.2/100,000 adults)	~1.15% of GBD adult estimate	MSEK-certified severe HL/deafness in adults
4a. Specialised treatment (adult)	Adult cochlear implantation	129 procedures (9.6/million adults)	~0.0056% of GBD adult estimate	Annual adult CI service response
4b. Specialised treatment (child)	Child cochlear implantation	394 procedures (57.1/million children)	—	Annual paediatric CI service response

Sources: This study (administrative); Bukenova et al. 2026 [12] (GBD-2021 Kazakhstan-specific modelled estimate). Percentages against GBD use the adult portion of the modelled estimate as the denominator: adult population 2025 (13,380,907) × ASPR 17,212/100,000 ≈ 2,303,000 estimated adult cases. Percentages are relative scale markers, not estimates of under-detection.

**Table 6:** International benchmarking of adult cochlear implantation rates.

Country	Year	CI Procedures (Total/Adult)	Adult CI per Million Adults	Source
Kazakhstan	2025	523/129	9.6	This study
Kazakhstan	2020	285/0	0.0	This study
Italy	2023	1595/~1117 (est)	~22 (est)	Ciminello et al. 2025 [17]
Germany	2022	2176/~1523 (est)	~22 (est)	Stöver et al. 2024 [18]
Japan	2023	1723/~1465 (est)	~14 (est)	Akazawa et al. 2025 [19]

Note: International adult subset estimates assume approximately 70% adult share for European programmes [17,18] and approximately 85% for Japan [19] based on published registry data; precise national adult-CI counts may differ.

## Data Availability

The data that support the findings of this study are available from the Republican Center for Electronic Health of the Ministry of Health of the Republic of Kazakhstan but restrictions apply to the availability of these data, which were used under license for the current study, and so are not publicly available. Data is however available from the corresponding author, upon reasonable request and with permission of the Ministry of Health of the Republic of Kazakhstan.

## Supplementary Materials

The following supporting information can be downloaded at: https://www.mdpi.com/article/10.3390/audiolres16040104/s1, Table S1: Full family of 20 Mann–Kendall tests with Benjamini–Hochberg FDR correction.
