# Supplementary material for: Hearing Impairment in Kazakhstan, 2020–2025: Dispensary Registration, Disability Certification, and Cochlear Implantation Response, with Reference to Global Burden of Disease 2021 Modelled Estimates
Source: Audiol Res. 2026 Jul 24;16(4):104. doi: 10.3390/audiolres16040104 (PMC13509522; doi:10.3390/audiolres16040104)
Supplement: Supplementary file 1 [file audiolres-16-00104-s001.zip › audiolres-4352876-supplementary.pdf]

Supplementary Table S1 — Full family of 20 Mann–Kendall tests with Benjamini–Hochberg FDR correction

This table lists all 20 indicators over which the Benjamini–Hochberg false-discovery-rate correction was applied. Indicators are ranked by raw p-value (ascending). BH-adjusted p-values (q) are calculated as q_i = p_i × m / rank_i, where m = 20, and are enforced monotone non-decreasing per the standard procedure. Values are computed for adults unless noted as (child).

#	Indicator	2020	2025	CAGR	MK τ	raw p	BH rank	FDR q	Sig.
1	H60–H95 adult, registered annual morbidity	231,209	278,897	+3.8%	+1.00	0.0028	1	0.016	✓
2	H60–H95 adult, first-time-in-life	126,513	167,674	+5.8%	+1.00	0.0028	2	0.016	✓
3	Total CI procedures (all ages)	285	523	+12.9%	+1.00	0.0028	3	0.016	✓
4	H65–H66 adult, active dispensary observation	6,871	2,245	−20.0%	−1.00	0.0028	4	0.016	✓
5	Disability registry, total hearing impairment cases	29,743	31,560	+1.2%	+1.00	0.0028	5	0.016	✓
6	Adult CI procedures (≥18)	0	129	n/a	+0.87	0.0166	6	0.037	✓
7	H60–H95 adult, active dispensary observation	21,840	19,881	−1.9%	−0.60	0.091	7	0.183	—
8	Child CI procedures (<18)	285	394	+6.7%	+0.60	0.091	8	0.183	—
9	H65–H66 adult, first-time-in-life	16,651	17,882	+1.4%	+0.47	0.188	9	0.319	—
10	Adult crude disability rate per 100,000	199.4	198.2	−0.1%	+0.33	0.260	10	0.371	—
11	H90–H91 child, first-time-in-life	3,990	4,148	+0.8%	+0.33	0.260	11	0.372	—
12	H65–H66 adult, registered annual morbidity	~40,300	~41,900	+0.8%	+0.33	0.260	12	0.433	—
13	Child crude disability rate per 100,000	73.0	75.4	+0.7%	+0.20	0.452	13	0.535	—
14	H90–H91 child, active dispensary observation	4,226	4,159	−0.3%	+0.07	0.548	14	0.535	—
15	H90–H91 child, registered annual morbidity	~10,500	~10,300	−0.4%	+0.07	0.548	15	0.535	—
16	Total crude disability rate per 100,000 (all ages)	159.6	155.6	−0.5%	+0.07	0.548	16	0.535	—
17	Adult disability rate per 100,000 (adjusted)	199.4	198.2	−0.1%	−0.07	0.707	17	0.535	—

#	Indicator	2020	2025	CAGR	MK τ	raw p	BH rank	FDR q	Sig.
18	Male-specific disability rate per 100,000	173.2	164.8	−1.0%	−0.20	0.707	18	0.535	—
19	Female-specific disability rate per 100,000	146.9	146.8	0.0%	+0.07	0.707	19	0.535	—
20	Male-to-female disability rate ratio	1.18	1.12	−1.0%	−0.07	1.000	20	1.000	—

Note. *Twenty indicators were subjected to Mann–Kendall trend testing over the six annual observations 2020–2025. Raw p-values are the two-sided Mann–Kendall p-values based on the exact distribution of τ under H_0 for n = 6; ties among identical p-values (e.g., τ = +1.00 or −1.00 all give p = 0.0028 under n = 6) are assigned successive BH ranks by convention. The Benjamini–Hochberg step-up procedure was applied with false-discovery-rate threshold α = 0.05. Indicators marked "✓" survived FDR correction. The seven headline indicators presented in Table 1 of the main text are ranks 1, 2, 4, 7, 9, 11, and 14 in this table.*

Reproducibility note. *The BH-adjusted q-values shown here reproduce the values reported in Table 1 (H60–H95 morbidity, H60–H95 first-time, H60–H95 active observation, H65–H66 first-time, H65–H66 active observation, H90–H91 first-time, H90–H91 active observation) exactly. Values shown with a leading tilde (~) for indicators 12 and 15 (H65–H66 and H90–H91 registered annual morbidity) are as reported to the analytic dataset; these were tested but not separately visualised in the main text.*
